# The influence of deuteration on the crystal structure of hybrid halide perovskites: a temperature-dependent neutron diffraction study of FAPbBr_3_

**DOI:** 10.1107/S2052520620002620

**Published:** 2020-03-20

**Authors:** Alexandra Franz, Daniel M. Többens, Frederike Lehmann, Martin Kärgell, Susan Schorr

**Affiliations:** a Structure and Dynamics of Energy Materials, Helmholtz-Zentrum Berlin für Materialien und Energie, Hahn-Meitner-Platz 1, Berlin, 14109, Germany; b Institute of Chemistry, University of Potsdam, Karl-Liebknecht-Str. 24-25, Potsdam, 14476, Germany; c Institute of Geological Sciences, Freie Universität Berlin, Malteserstr. 74-100, Berlin, 12249, Germany

**Keywords:** hybrid perovskite, FAPbBr_3_, deuteration, neutron powder diffraction, crystal structure

## Abstract

This paper discusses the full structural solution of the hybrid perovskite formamidinium lead tribromide (FAPbBr_3_) and its temperature-dependent phase transitions in the range from 3 K to 300 K using neutron powder diffraction and synchrotron X-ray diffraction. Special emphasis is put on the influence of deuteration on formamidinium, its position in the unit cell and disordering in comparison to fully hydrogenated FAPbBr_3_. The temperature-dependent measurements show that deuteration critically influences the crystal structures, *i.e.* results in partially-ordered temperature-dependent structural modifications in which two symmetry-independent molecule positions with additional dislocation of the molecular centre atom and molecular angle inclinations are present.

## Abstract 2

Deuteration severely influences all temperature-dependent structural modifications in the hybrid perovskite FAPbBr_3_. It leads to partially-ordered temperature-dependent structural modifications in which two symmetry-independent molecule positions with additional dislocation of the molecular centre atom and molecular angle inclinations are present.

## 1. Introduction   

**Figure 1:**
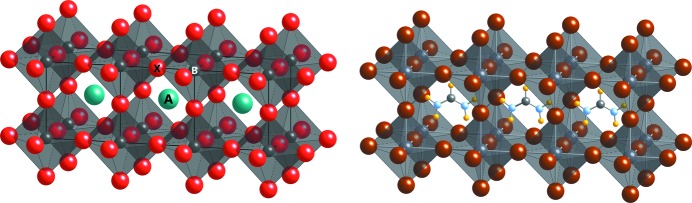
Left: inorganic ‘classic’ oxide perovskite as an example for *ABX*_3_ stoichiometry: visualization of the cubic aristotype with the *A* cation centred in the cuboctahedral void of corner-sharing *BX*_6_ octahedra. Right: hybrid perovskite structure visualization with formamidinium as the *A* cation and with *X* as a halide ion.

The aristotype of the perovskite-type crystal structure is defined as *A* ^[XII]^ *B* ^[VI]^ *X*_3_ stoichiometry with a corner-sharing network of octahedra in which the *A* cation is cuboctahedrally surrounded by oxygen atoms. The tetravalent *B* cation builds together with oxygen the aforementioned corner-sharing octahedral network. Hybrid perovskites show a corner-sharing network as well but the anion is a group 17 element: bromine, iodine or chlorine. Eponymous for hybrid perovskites is the replacement of the large inorganic *A* cation by an organic molecule *e.g.* methyl­ammonium [CH_3_NH_3_]^+^ (abbreviated as MA) or formamidinium [H_2_NCH=NH_2_]^+^ (FA), forming an organic–inorganic hybrid perovskite (see right-hand view in Fig. 1 ▸). At 2.53 Å the radius of the formamidinium molecule (Kieslich *et al.*, 2014 ▸) is one-and-a-half times that of the inorganic *A* cation which results in an increase in the hybrid perovskite (∼6 Å) cubic lattice parameter compared to inorganic perovskite (∼4 Å).

Different temperature-dependent crystal structures in perovskites can be visualized by the Bärnighausen family tree (Bärnighausen, 1980 ▸). This family tree describes in descending order and proceeding from the aristotype (space group 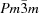) crystallographic group–subgroup relations of the hettotype structures.

These derived space groups (with lower symmetry) show different kinds of distortions (see Megaw, 1973 ▸). Symmetry lowering, resulting from tilting of the *BX*_6_ octahedra, is also discussed by Glazer (1972 ▸), Woodward (1997 ▸), Lufaso & Woodward (2001 ▸) and Bock & Müller (2002 ▸). Commonly used is the three-letter-notation developed by Glazer in which the magnitude of tilting of the coordination octahedra around the [100], [010] and [001] directions of the perovskite unit cell relative to the Cartesian axes is specified. A superscript defines whether the adjacent layers rotate in the same (+) or in the opposite (−) direction.

Deuteration is often applied in the study of hydrogen-containing structures by neutron diffraction, as the very high incoherent scattering length of ^1^H results in high background noise (Sears, 2006 ▸). Although it was assumed that the deuteration in most cases does not influence the crystal structure (Fisher & Helliwell, 2008 ▸), it has been reported by Shi *et al.* (2018 ▸) and Whitfield *et al.* (2016 ▸) that small changes in bond length and angles can occur. Merz & Kupka (2015 ▸) report on changes in the geometric arrangements of molecules in a crystal framework after the replacement of protium (H) by deuterium (D). Furthermore, Harwell *et al.* (2018 ▸) report on changes in physical properties caused by deuteration of the FA molecule. Contradictory reports are given by Dunitz & Ibberson (2008 ▸) and Fortes & Capelli (2018 ▸) who discuss the influence of deuteration on the temperature-dependent unit-cell volume. Since hydrogen and deuterium differ in mass and spin (Shi *et al.*, 2018 ▸) and, furthermore, deuterium shows a smaller effective (van der Waals) radius (Dunitz & Ibberson, 2008 ▸), it is reasonable to assume an influence of the deuteration on the crystal structure of this hybrid perovskite.

In this paper we discuss the full structural solution for deuterated (D_4_) and hydrogenous HC(NH_2_)_2_PbBr_3_ (abbreviated as FAPbBr_3_) over the temperature range from 3 to 300 K and the impact of deuteration on the crystal structure.

## 2. Experimental procedure   

### 2.1. Synthesis of FAPbBr_3_   

Powder samples were synthesized using different routes [after Saidaminov *et al.* (2015 ▸) and Baikie *et al.* (2013 ▸)] for the D_4_ and hydrogenous samples. Hydrogenous FAPbBr_3_ powder was synthesized from stoichiometric mixtures of FABr (99.99% from Ossila) and PbBr_2_ (98+%, extra pure, from Arcos Organics) in di­methyl­formamide (DMF, 99.8%, Roth) and homogenized overnight at room temperature, followed by evaporation of the solvent at 75°C, yielding 100% FAPbBr_3_.

### 2.2. Synthesis of (D_4_)-FAPbBr_3_   

16.7 g (0.160 mol, 1 equiv.) of formamidinium acetate was dissolved in 25 ml milliQ water (18.2 MΩ) in a 100 ml two-neck flask equipped with reflux condenser, dropping funnel and magnetic stirring bar. The reaction mixture was cooled in an ice bath. Subsequently, 4.5 ml hydro­bromide acid (HBr in 47% water, 0.176 mol, 1.1 equiv.) in 10 ml milliQ water was added under constant stirring. Then, HBr was added to the reaction mixture. This mixture was heated to reflux for around one hour. Afterwards water and acetic acid were distilled in a rotary evaporator until a white powder occurred. Subsequently, another 4.5 ml of hydro­bromic acid in 10 ml milliQ water was added and distilled in the rotary evaporator until 19.87 g (99.35%) formamidinium bromide, as a white crystalline powder resulted.

In a 50 ml flask with stirring bar and a stopper, 8 g of formamidinium bromide was dissolved in *ca* 25 ml of deuterium oxide. Additionally 1–2 ml (0.064 mol) of deuterium bromide in D_2_O were added. After one hour of stirring, the deuterium oxide was removed by rotary evaporation. This procedure was repeated three times, yielding 7.95 g formamidinium bromide D_4_. Afterwards the dry product was used in the following steps without further purification. The final reaction step was similar to the aforementioned synthesis procedure for the hydrogenous FAPbBr_3_ only exchanging FABr with (D_4_)-FABr. The samples were stored under N_2_ to avoid potential degradation by oxygen and humidity.

### 2.3. Neutron powder diffraction and synchrotron X-ray powder diffraction   

For a reliable detection of hydrogen/deuterium positions and to distinguish between C and N, neutron powder diffractograms were collected at the fine-resolution powder diffractometer E9 (FIREPOD) at the BER II neutron source at Helmholtz-Zentrum Berlin (HZB) (Franz & Hoser, 2017 ▸). A deuterated (D_4_) and a hydrogenated FAPbBr_3_ sample were placed in 6 mm diameter vanadium cans and measured in a temperature range of 3–300 K using a dry cryostat (TROK). A wavelength of λ = 1.7982 (1) Å (511-Ge) was applied.

X-ray powder diffraction data in the range of 5°< 2θ < 134° were collected for FAPbBr_3_ at the diffraction end station of KMC-2 beamline (BESSY II, HZB; Többens & Zander, 2016 ▸) using a radiation energy of 8048 eV [λ = 1.5406 (1) Å]. Additionally, for low-temperature experiments a modified Gifford–McMahon (GM) closed-cycle cryocooler, in-house label CCR-XRD, configured with a double Kapton cupola and helium exchange gas was used (HZB, 2018 ▸). For an exact determination of the phase transition temperatures selected-region powder diffractograms (25°< 2θ < 35°) in the range from 20 to 285 K in 1 K steps were additionally taken.

### 2.4. Analysis   

#### 2.4.1. DFT calculations   

As part of the structure determination process, in order to evaluate potential structure candidates, a number of Density Functional Theory (DFT) calculations were conducted on different fully ordered arrangements of the formamidinium cation in the ortho­rhombic structure, assuming space group *Pnma* and subgroups thereof. Calculations were performed from first principles with the program *CRYSTAL14* (Dovesi *et al.*, 2014 ▸) using 3D-periodic DFT with Gaussian basis sets and the PBE0 Hamiltonian (Adamo & Barone, 1999 ▸). Basis sets and other computational parameters were as used previously by us (Schuck *et al.*, 2018 ▸). The crystal structures were allowed to fully relax upon energy minimization. The molecule geometry from the optimization yielding the lowest energy was used as a rigid unit in both structure determination and refinement, and was kept unchanged in the final results (see Table S1).

#### 2.4.2. Structure analysis and Rietveld refinement   

The structure determination was performed by parallel tempering using *FOX* (Favre-Nicolin & Cerný, 2002 ▸; http://objcryst.sourceforge.net). During this process, the positions of Pb, Br, FA were restrained to stay close to their sites within the perovskite structure. Geometry restraints of the molecule were treated as flexible using the software’s default values for the initial search, followed by strict idealization and subsequent optimization as a rigid body. Structure solution calculations were performed independently for D_4_ and hydrogenated forms.

For tetragonal and orthorhombic perovskite structures, multiple attempts were conducted to improve the results using either symmetry reduction to a subgroup or multiple independent formamidinium molecules. None of these resulted in significant improvements.

Subsequent structure refinement applying the Rietveld method was carried out with the *FullProf Suite* (Rodríguez-Carvajal, 1993 ▸), using the rigid-body option of the software for the formamidinium cation. Compared with the structure determination software *FOX*, this allowed an enhanced modelling of the atomic displacement with individual isotropic and anisotropic displacement parameters, and TLS-displacement (translation-libration-screw rotation) of the molecule (Schomaker & Trueblood, 1968 ▸). It was found, however, that anisotropic and TLS parameters correlated strongly with the molecular position, without resulting in significant improvements of the refinement. In our experience this is characteristic for structures with high disorder related to small deviations of the structure from a broken higher symmetry. Hence, only a simple displacement model was selected, with isotropic displacement parameters for all atoms and a uniform displacement parameter for all atoms of the formamidinium cation.

At temperatures of 10, 240 and 300 K, neutron diffraction data were collected for both (D_4_)-FAPbBr_3_ and FAPbBr_3_. It was neither possible to find a structure allowing a joint refinement of these data sets with a single set of atomic positions nor to describe the diffraction pattern of the deuterated sample with the structure parameters refined from the hydrogenated one and vice versa. The resulting structural differences are highly significant and very strong, indicating that the structural differences between (D_4_)-FAPbBr_3_ and FAPbBr_3_ discussed here are not negligible details.

## 3. Results   

### The cubic *Pm* 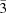 *m* structure at 300 K   

3.1.

The overall room-temperature structures of (D_4_)-FAPbBr_3_ and FAPbBr_3_ were found to be similar to studies performed by Schueller *et al.* (2018 ▸) and show the Glazer notation *a* ^0^ *a* ^0^ *a* ^0^. No octahedral tilting is present.

In a refinement from laboratory X-ray powder diffraction data, Hanusch *et al.* (2014 ▸) placed the central carbon of the FA molecule as the rotational centre at the 1*b* Wyckoff position at (½, ½, ½) in the centre of the cuboctahedral cage. Nitro­gen was placed twelvefold disordered at (*y*, *y*, ½) with *y* = 0.3363 (5), with both nitro­gen atoms in the molecule represented by the same Wyckoff position. This simplifying approach restricts the N—C—N angle to a reasonable value of 120°.

From single-crystal X-ray diffraction data, Govinda *et al.* (2018 ▸) refined this model by allowing both atoms to deviate from their high-symmetry site, with C at 6*f* (*x*, ½, ½) and N at 24*l* (*x*, *y*, ½). This refinement places the carbon 0.42 (1) Å away from the centre of the cage, but does not fundamentally change the model. In both cases, the high site symmetry 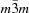 of 1*b* results in a sphere of disordered nitro­gen atoms around a carbon atom or a small sphere of disordered carbon atoms. Hydrogen atoms are not included in either model.

Our approach, using rigid-body modelling to restrict the number of free parameters, as a matter of principle places every atom of the molecule on a low symmetry, general Wyckoff site 48*n* (*x, y, z*). This of course results again in embedded spheres of carbon, nitro­gen and hydrogen. However, in contrast to previously published models the distribution of scattering power density within the spheres must be consistent with intramolecular geometry. This, together with the non-negligible scattering power of hydrogen, allows for a determination of the real positioning of the individual molecules underlying the disordered distribution.

Our results for FAPbBr_3_ are in good agreement with the above-mentioned results. Carbon is located almost in the middle of the cubic unit cell, at 0.49 Å from the centre, and very close to the positions found by Govinda *et al.* (2018 ▸). However, where the older models restrict the nitro­gen to a single sphere, we found the two independent nitro­gen atoms to occupy very different positions. One, 1.03 Å from the centre approximately at (*y*, *y*, ½), conforms to the position found in the previous studies. The other, 1.36 Å from the centre and approximately at (*x*, ½, ½), is a newly identified position. Both positions place nitro­gen at approximately 3.2 Å from the bromine anion, in positions to form hydrogen bonds. Overall, this structure is not significantly different from the published structures, but described by a more detailed model.

**Figure 2:**
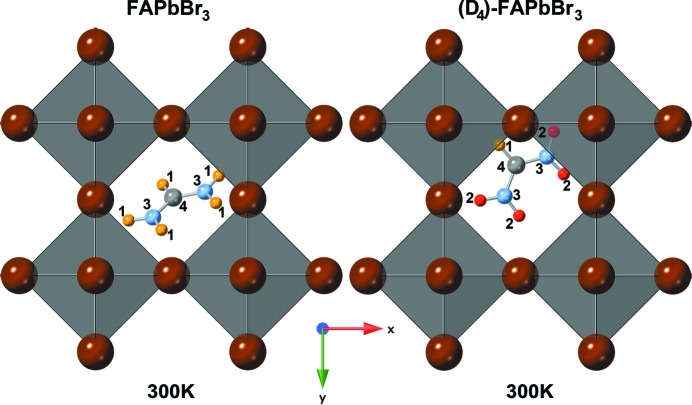
Octahedral network of FAPbBr_3_ and (D_4_)-FAPbBr_3_ at 300 K (space group 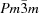). Displayed here is one FA position out of 48; left: FAPbBr_3_ unit cell with PbBr_6_ octahedra and the inclined FA molecule in the centre; Right: (D_4_)-FAPbBr_3_ with its FA molecule strongly dislocated from the unit-cell centre; the atoms are number and colour coded as follows: hydrogen – 1, yellow; deuterium – 2, red; nitro­gen – 3, light blue; carbon – 4, light grey, lead (octahedra) – grey; bromine – brown (edges of octahedra).

The situation is different for (D_4_)-FAPbBr_3_ (see Fig. 2 ▸). In the deuterated compound the FA cation is found to be heavily decentred, with the central carbon atom at more than 2 Å distance from the 2*b* site and one of the nitro­gen atoms close to it. This displacement is accompanied by very high Debye–Waller factors in particular of the FA cation. Pb and Br also have higher Debye–Waller factors than in the hydrogenated compound. A possible reason for this high displacive disorder might be that the higher mass of the deuterium hinders the rotation of the cation and thus the breaking of the hydrogen bonds necessary for this rotation. The resulting stronger average hydrogen bond might pull the cation out of its average position in the centre of the cage. A problematic aspect of the observed structure is that it places one deuterium too close to Br^−^, at a distance of only 1.73 Å. This, however, can be explained by geometric effects that make the distance between the average atom positions obtained by diffraction appear shorter than the real local distances. The low-temperature structure refinement shows that the displacement of Br from its high-symmetry position in the cubic structure can be more than 0.4 Å; this is in agreement with the r.m.s. displacement from the Debye–Waller factor. In addition the strong displacement of the cation in the direction lateral to the Br⋯D—N hydrogen bond also means that real local Br⋯D distances are much higher. In the following discussion of the low-temperature structures we will see that this motif of a highly displaced FA^+^ cation is present in all modifications of the deuterated compound.

### 3.2. The tetragonal *P*4/*mbm* structure at 240 and 180K   

**Figure 3:**
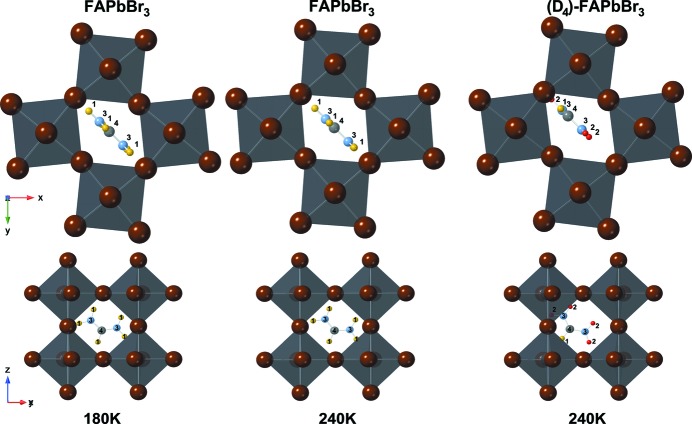
Octahedral network of FAPbBr_3_ at 180 and 240 K (left and middle) and (D_4_)-FAPbBr_3_ (right) shown along [001] (top) and [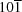] (bottom); the crystal structures adopt space group *P*4/*mbm* with in-plane tilted octahedra around the *c* axis with the molecular distribution at 240 K and 180 K in [110]; in (D_4_)-FAPbBr_3_, the FA molecule is dislocated from its central position in the cuboctahedral void; the atoms are number and colour coded as follows: hydrogen – 1, yellow; deuterium – 2, red; nitro­gen – 3, light blue; carbon – 4, light grey, lead (octahedra) – grey; bromine – brown (edges of octahedra).

The neutron data refinement of FAPbBr_3_ at 240 K and 180 K, and (D_4_)-FAPbBr_3_ at 240 K yield the tetragonal space group *P*4/*mbm* (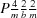) in which the octahedral network tilts in-plane around the *c* axis (*a* ^0^ *a* ^0^ *c* ^+^) (see Fig. 3 ▸). In this space group, the centre of the cage at site 2*c* has local symmetry *mmm*. As this symmetry is higher than the molecule symmetry of *mm*2, at least twofold disorder is inevitable. However, a significant tilt of the molecule breaks symmetry even further, resulting in a fourfold disorder with formamidinium on site 8*j* with site symmetry *m*. The placement of the molecule at this special site, where the mirror plane of the molecule coincides with the mirror plane of the space group, was not purported by the structural model, it resulted from the Rietveld refinement without significant deviations. While the preceding is valid regardless of deuteration state, the structures differ in the placement of the molecule. In the hydrogenated form, displacement from the centre of the cage is again small. This results in the formation of nearly symmetric, but weak N—H⋯Br hydrogen bonds with two opposing edges of the cage. In (D_4_)-FAPbBr_3_, the molecular centre atom carbon is strongly shifted out of the centre position. Consequently, only one N—D⋯Br hydrogen bond is formed, which is shorter. The position and orientation of the formamidinium cation in the hydrogenated form is very similar to the one underlying the disordered distribution in the cubic phase.

### 3.3. The orthorhombic *Pnma* structure at 140, 10 and 3 K   

The measurements taken at the BERII neutron source were at 10 K for FAPbBr_3_, and at 140, 10 and 3 K for (D_4_)-FAPbBr_3_. Due to the extensively long measurement times for the hydrogenous sample (to reach satisfying neutron counts statistics of the powder pattern) only one measurement in the stability region of the orthorhombic phase could be performed.

**Figure 4:**
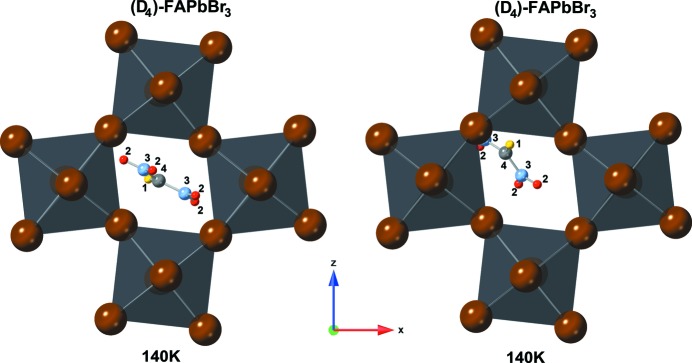
Visualization of the (D_4_)-FAPbBr_3_ in which the refinement resulted in two symmetry-independent molecular positions: molecule 1 (left) is diagonally located in the Br-lozenge with the molecular centre atom C in the middle; molecule 2 (right) is strongly off-centred towards the PbBr_6_ network. The atoms are number and colour coded as follows: hydrogen – 1, yellow; deuterium – 2, red; nitro­gen – 3, light blue; carbon – 4, light grey, lead (octahedra) – grey; bromine – brown (edges of octahedra).

**Figure 5:**
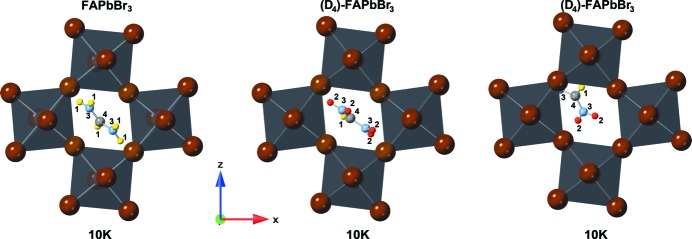
Visualization of octahedral networks of FAPbBr_3_ (left) and (D_4_)-FAPbBr_3_ (middle and right) at 10 K. (D_4_)-FAPbBr_3_ shows two symmetry-independent molecular positions in which the second molecule is strongly off-centred from the Br-lozenge centre. The atoms are number and colour coded as follows: hydrogen – 1, yellow; deuterium – 2, red; nitro­gen – 3, light blue; carbon – 4, light grey, lead (octahedra) – grey; bromine – brown (edges of octahedra).

The low-temperature phases (see Figs. 4 ▸, 5 ▸ and 6 ▸) of FAPbBr_3_ adopt the orthorhombic crystal system with space group *Pnma * (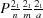). The local symmetry at the centre of the cage is *m_b_*, a mirror plane perpendicular to *b*. This would allow for a fully ordered structure with the planar FA molecule inside the mirror plane. This is not realized. In the hydrogenated forms the planar FA molecule is oriented nearly exactly perpendicular to *m_b_* (see Fig. 5 ▸, left). This results in twofold disorder, with 0.5:0.5 partial occupation of symmetrically equivalent orientations. The placement of the FA cation close to the centre of the cage and its orientation are very similar to the positioning underlying the distribution in the tetragonal form of the compound.

**Figure 6:**
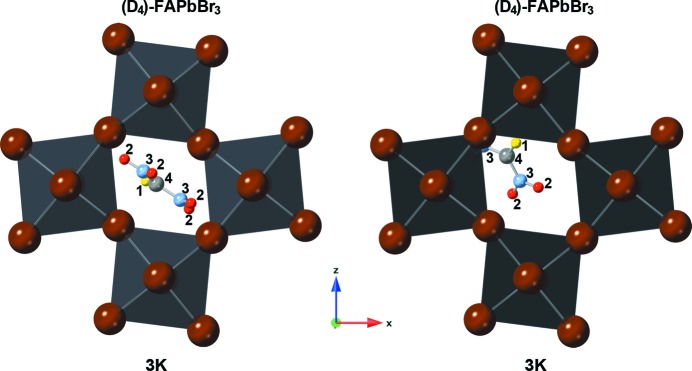
(D_4_)-FAPbBr_3_ network at 3 K with visualization of the two symmetry-independent molecular positions and their different inclination angles. The atoms are number and colour coded as follows: hydrogen – 1, yellow; deuterium – 2, red; nitro­gen – 3, light blue; carbon – 4, light grey, lead (octahedra) – grey; bromine – brown (edges of octahedra).

The structures of deuterated (D_4_)-FAPbBr_3_ could not be described satisfactory with only one independent FA molecule. It was necessary to split it into two independent ones, resulting in an overall fourfold disorder (see Fig. 5 ▸, middle and right; Fig. 6 ▸). The distribution of the cation over these two independent sites is stable, refining to occupation ratios of 0.58 (1): 0.42 (1) without any significant change over the whole temperature range (see Fig. 6 ▸). The higher occupied position is once again similar to the one observed in the respective, D_4_ in this case, tetragonal form. The cation is in an orientation lateral to the *m_b_* plane, but shifted out of the centre of the cage and tilted, so that a single N—D⋯Br hydrogen bond can form. The cations at the lower occupied site, on the other hand, are shifted so far from the centre that one ND_2_ group extends into the lozenge spanned by bromine that forms the window between adjacent cages.

### 3.4. Temperature-dependent phase transitions   

**Figure 7:**
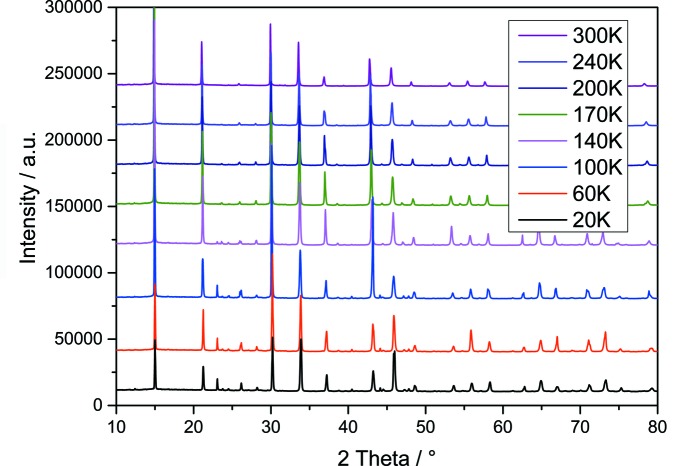
Overview of synchrotron powder diffraction data of FAPbBr_3_ taken from 20 K to 300 K in 30–60 K steps.

**Figure 8:**
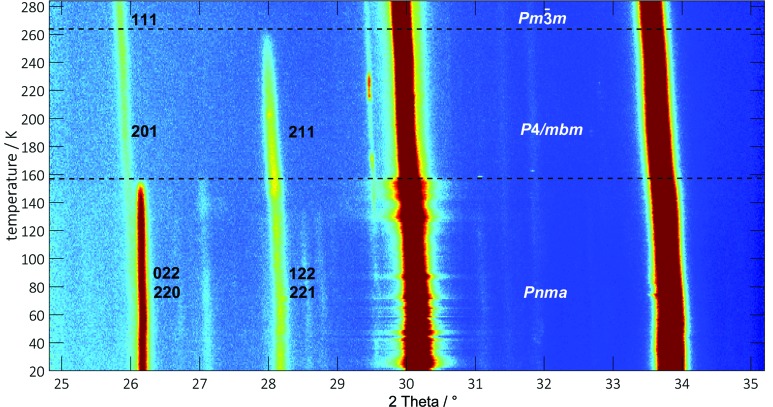
Synchrotron X-ray diffraction contour colour plot of a selected 2θ region, collected using a ramp rate of 1 K and showing the temperature-dependent phase transition of FAPbBr_3_ from *Pnma* to *P*4/*mbm* at 157 K (lower dashed black line) and from *P*4/*mbm* to 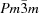 at 264 K (upper dashed black line).

In the synchrotron diffraction pattern overview (see Fig. 7 ▸) it can be clearly seen that the difference in the powder pattern is fairly small and the evidence-giving region is between 25° < 2θ < 35°. Thus, regions of interest scans were taken in 1 K steps and the phase transition temperatures could be determined. The 220/022 superlattice reflections show the orthorhombic–tetragonal phase transition at 157 K. The orthorhombic 221/122 superlattice reflections vanish at 264 K and as a consequence, mark the tetragonal–cubic phase transition (see Fig. 8 ▸).

**Figure 9:**
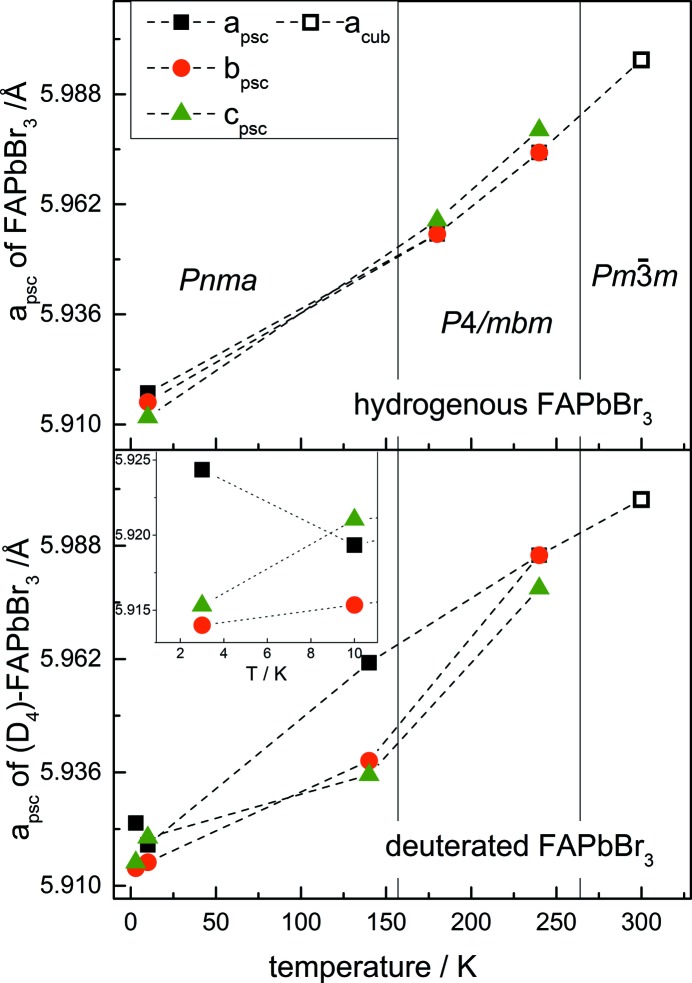
Evolution of the lattice parameters in terms of increasing temperature of hydrogenous (upper view) and deuterated (lower view) FAPbBr_3_. The inset in the bottom figure displays the zoomed region between 0 and 10 K. For a better comparability the pseudocubic lattice parameters are displayed. The error bars are smaller than the symbol sizes and the dashed lines are only a guide for the eye. The pseudocubic *a* lattice parameter (black squares) in (D_4_)-FAPbBr_3_ shows an unusual thermal behaviour shortly before the phase transition from *Pnma* to *P*4/*mbm*.

Fig. 9 ▸ shows the temperature dependence of the lattice parameters of both hydrogenous FAPbBr_3_ and (D_4_)-FAPbBr_3_ as determined by the refinement of the neutron diffraction data. For a better comparability all lattice parameters are shown as pseudocubic (indexed as psc) with *a*, *c*_psc_ = 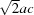 and *b*_psc_ = *b*/2. The orthorhombic, tetragonal and cubic lattice parameters are shown in Table S1. The thermal expansion of the pseudocubic lattice parameters of the hydrogenous sample follow – as expected – a clear trend up to 300 K. In contrast, the lattice parameter *a* of (D_4_)-FAPbBr_3_ shows an unusual behaviour shortly before the phase transition from *Pnma* to *P*4/*mbm* (Fig. 9 ▸, bottom view).

**Figure 10:**
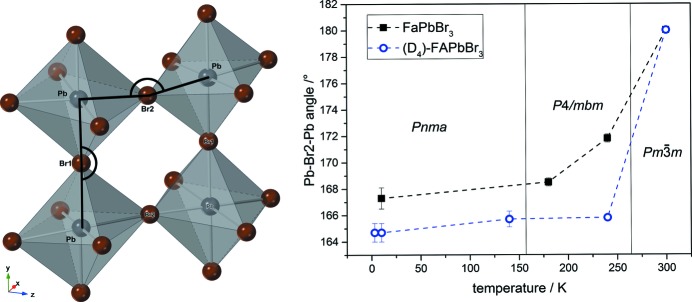
Comparison of the tilt angle Pb—Br2—Pb in FAPbBr_3_ and (D_4_)-FaPbBr_3_. The spatial extent caused by the two molecular positions leads to an increased tilting of the octahedral network. The dashed lines are only a guide for the eye.

The connected PbBr_6_ octahedra are defined by the Pb—Br1—Pb and Pb—Br2—Pb angles (see Fig. 10 ▸, left-hand-side; the FA molecule is not displayed for clarity). With increasing temperature, these angles increase and converge to reach 180° at room temperature, leading to an arrangement of the octahedra as shown in Fig. 2 ▸ for FAPbBr_3_ and (D_4_)-FAPbBr_3_.

Glazer notation of octahedral tilting in *Pnma* is *a* ^−^ *b* ^+^ *a* ^−^ with an out-of-phase tilting around the cubic *a* axes and an in-phase tilting around the cubic *b* axis ([001]_cub_ = [101]_orth_, [010]_cub_ = [010]_orth_ and [100]_cub_ = [10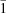]_orth_). The latter is shown in Fig. 10 ▸ (right) as Pb—Br2—Pb angle. In contrast to hydrogenous FAPbBr_3_, the tilt angle in (D_4_)-FAPbBr_3_ in the range from 3 to 240 K is lower and, furthermore, does not show a significant increase up to 240 K. The two symmetry-independent molecular sites which are present in all deuterated low-temperature modifications crystallizing in space group *Pnma* require more space in the voids between the octahedra. To compensate for this, the octahedral network is forced to tilt further. Since the FA molecule is located in the *ac* plane the lattice parameters are influenced by this as well.

**Figure 11:**
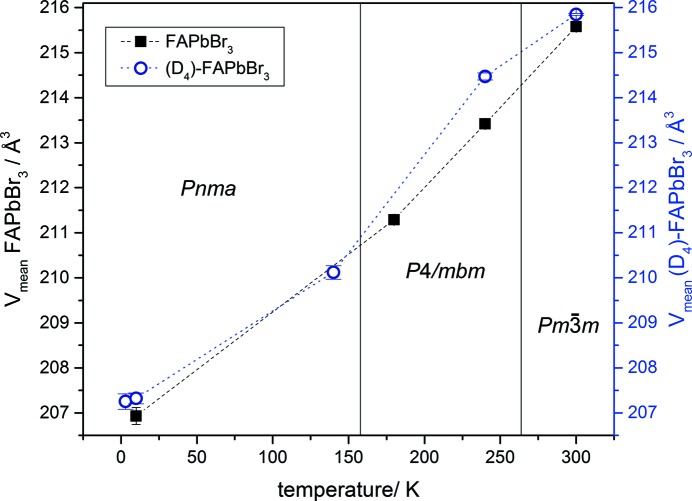
Comparison of mean unit-cell volumes of FAPbBr_3_ (black squares) and (D_4_)-FAPbBr_3_ (blue open circles). (D_4_)-FAPbBr_3_ shows the larger unit cell, compared to the FAPbBr_3_. The dashed lines are only a guide for the eye.

Fig. 11 ▸ displays the mean unit-cell volumes of hydrogenous and deuterated FAPbBr_3_ from 3 to 300 K (calculated from the pseudocubic lattice parameters and chosen due to the enhanced comparability of the values). The graph shows the increased unit-cell volume of (D_4_)-FAPbBr_3_ due to deuteration. The unusual thermal behaviour of the *a* lattice parameters at 140 K (see Fig. 9 ▸) is reflected in the value for the volume of (D_4_)-FAPbBr_3_ and is in the range of the value of the hydrogen-containing samples.

## 4. Summary   

The strong influence of deuteration on the crystal structure of FAPbBr_3_ was demonstrated by a detailed neutron diffraction investigation over a wide temperature range. By deuteration of FAPbBr_3_ different partially-ordered crystal structures and increased lattice parameters have been observed. The deuterated FA molecule shows two symmetry-independent sites instead of one, leading to an increased tilt angle between the corner-sharing PbBr_6_ octahedra. (D_4_)-FAPbBr_3_ shows an additional molecular disordering in the *Pnma* region caused by a strong off-centre shift of the molecular position between the corner-sharing octahedra.

## Supplementary Material

Crystal structure: contains datablock(s) global, FAPbBr3_1H_10K, FAPbBr3_D4_10K, FAPbBr3_D4_140K, FAPbBr31H180K, FAPbBr3D43K, FAPbBr31H240K, FAPbBr3D4-240K, FAPbBr3_1H_300K, FAPbBr3_D4_300K. DOI: 10.1107/S2052520620002620/ra5076sup1.cif

Tables S1 and S2. DOI: 10.1107/S2052520620002620/ra5076sup11.pdf

Rietveld powder data: contains datablock(s) FAPbBr3_1H_10K. DOI: 10.1107/S2052520620002620/ra5076FAPbBr3_1H_10Ksup2.rtv

Rietveld powder data: contains datablock(s) FAPbBr_D4_10K. DOI: 10.1107/S2052520620002620/ra5076FAPbBr3_D4_10Ksup3.rtv

Rietveld powder data: contains datablock(s) FAPbBr3_D4_140K. DOI: 10.1107/S2052520620002620/ra5076FAPbBr3_D4_140Ksup4.rtv

Rietveld powder data: contains datablock(s) FAPbBr3_1H_180K. DOI: 10.1107/S2052520620002620/ra5076FAPbBr3_1H_180Ksup5.rtv

Rietveld powder data: contains datablock(s) FAPbBr3_D4_3K. DOI: 10.1107/S2052520620002620/ra5076FAPbBr3_D4_3Ksup6.rtv

Rietveld powder data: contains datablock(s) FAPbBr3_1H_240K. DOI: 10.1107/S2052520620002620/ra5076FAPbBr3_1H_240Ksup7.rtv

Rietveld powder data: contains datablock(s) FAPbBr3_D4_240K. DOI: 10.1107/S2052520620002620/ra5076FAPbBr3_D4_240Ksup8.rtv

Rietveld powder data: contains datablock(s) FAPbBr3_1H_300K. DOI: 10.1107/S2052520620002620/ra5076FAPbBr3_1H_300Ksup9.rtv

Rietveld powder data: contains datablock(s) FAPbBr3_D4_300K. DOI: 10.1107/S2052520620002620/ra5076FAPbBr3_D4_300Ksup10.rtv

CCDC references: 1987741, 1987742, 1987743, 1987744, 1987745, 1987746, 1987747, 1987748, 1987749
