# Supplementary material for: The influence of deuteration on the crystal structure of hybrid halide perovskites: a temperature-dependent neutron diffraction study of FAPbBr3
Source: Acta Crystallogr B Struct Sci Cryst Eng Mater. 2020 Mar 20;76(Pt 2):267–74. doi: 10.1107/S2052520620002620 (PMC7137785; doi:10.1107/S2052520620002620)
Supplement: Supplementary file 2 [file b-76-00267-sup11.pdf]

STRUCTURAL SCIENCE
CRYSTAL ENGINEERING
MATERIALS

Volume 76 (2020)

Supporting information for article:

The influence of deuteration on the crystal structure of hybrid halide perovskites: a temperature dependent neutron diffraction study of FAPbBr3

Alexandra Franz, Daniel M. Többens, Frederike Lehmann, Martin Kärgell and Susan Schorr

Table S1 Geometry of the FA-cation

Distances / Å	
C-N	1.303
C-H	1.082
N-H/D	1.014
Angles / °	
N-C-N	124.58
N-C-H	117.71
C-N-H/D	119.55 (outside)
	121.10 (inside)
H/D-N-H/D	119.35

Table S2 Temperature dependent lattice parameters, unit cell volumes, octahedral bond length and Pb-Br-Pb angles of the hydrogenous and deuterated $FAPbBr_3$

Coordination geometry in $FAPbBr_3$ and (D_4)-$FAPbBr_3$

Temp./ K	Space group	Lattice parameters/ Å	Unit cell volume/ Å^3	*d* Pb-Br/ Å	∡Pb-Br-Pb/ °	∡Br-Pb-Br*/ °
			($HC(NH_2)_2PbBr_3$)			
10	*Pnma*	*a* = 8.3685(13)	827.71(19)	2.977(2) 2x	166.83(6) 2x	80.5(10)
		b = 11.8306(9)		2.99(3) 2x	167.3(8) 4x	89.5(10)
		c = 8.3604(12)		2.96(3) 2x		90
180	*P4/mbm*	*a* = 8.4216(4)	422.58(5)	2.9791(3) 2x	180 2x	90
		c = 5.9582(5)		2.993(9) 4x	168.5(3) 4x	
240	*P4/mbm*	*a* = 8.4488(3)	426.83(4)	2.9898(3) 2x	180 2x	90
		c = 5.9795(5)		2.995(10) 4x	171.8(3) 4x	
300	*Pm-3m*	*a* = 5.99609(19)	215.58(1)	2.9981(1) 6x	180 6x	90

Temp./ K	Space group	Lattice parameters/ Å	Unit cell volume/ Å^3	*d* Pb-Br/ Å	∡Pb-Br-Pb/ °	∡Br-Pb-Br*/ °
			**($HC(ND_2)_2PbBr_3$)**			
3	*Pnma*	*a* = 8.3783(10)	829.01(17)	2.9581(6) 2x	176.92(3) 2x	86.4(9)
		b = 11.8280(13)		3.04(3) 2x	164.7(7) 4x	84.4(9)
		c = 8.3655(11)		2.93(3) 2x		90
10	*Pnma*	*a* = 8.3712(3)	829.30(12)	2.9586(5) 2x	177.16(2) 2x	86.0(8)
		b = 11.8307(9)		3.04(3) 2x	164.7(7) 4x	84.4(8)
		c = 8.3736(10)		2.93(3) 2x		90
140	*Pnma*	*a* = 8.4303(9)	840.47(15)	2.9728(10) 2x	174.48(3) 2x	84.0(8)
		b = 11.8773(12)		3.00(2) 2x	165.7(6) 4x	87.3(8)
		c = 8.3939(8)		3.00 (2) 2x		90
240	*P4/mbm*	*a* = 8.4652(8)	428.39(8)	2.9891(4) 2x	180 2x	90
		c = 5.9782(8)		3.016(8) 4x	165.8(2) 4x	
300	$Pm\bar{3}m$	*a* = 5.9986(3)	215.85(2)	2.9993(2) 6x	180 6x	90

Due to symmetry limitations, only ∡Br1-Pb-Br2 in *Pnma* can deviate from 90°; of paired angles >90° and <90° only the latter is listed.
